# MC3 Mucoepidermoid carcinoma cell line enriched cancer stem-like cells following chemotherapy

**DOI:** 10.3892/ol.2014.1902

**Published:** 2014-02-21

**Authors:** LOUQIANG ZHANG, LONGJIANG LI, YIN WANG, YING LIU, CHUNJIE LI

**Affiliations:** 1 Department of Stomatology, Tianjin Medical University General Hospital, Tianjin 300052, P.R. China; 2 State Key Laboratory of Oral Diseases, West China Hospital of Stomatology, Sichuan University, Chengdu, Sichuan 610041, P.R. China; 3 Department of Head and Neck Oncology Surgery, West China Hospital of Stomatology, Sichuan University, Chengdu, Sichuan 610041, P.R. China

**Keywords:** cancer stem-like cells, mucoepidermoid carcinoma, octamer-binding transcription factor 4, cluster of differentiation 44, 5-fluorouracil

## Abstract

Mucoepidermoid carcinoma (MEC) is common in human salivary glands. Surgery is the preferred treatment method for MEC and chemotherapy is often administered following surgery as an adjuvant cancer treatment; however, chemotherapy does not completely prevent tumor recurrence. Emerging evidence has indicated the existence of cancer stem-like (CSL)-cells in tumors. CSL-cells are important in the development, invasion and drug resistance of carcinomas. The present study aimed to investigate whether chemotherapy enriched the CSL-cells in the MEC cell line of MC3 using 5-fluorouracil (5-Fu). The MC3 cells were treated with 5-Fu, which enhanced the spherogenesis and vitality of the cells and upregulated the pluripotency gene, octamer-binding transcription factor 4. Side population analysis demonstrated that the proportion of CSL-cells also increased. These findings showed that compared with other types of cancer cells, chemotherapy was unable to effectively kill the CSL-cells resulting in an enriched CSL-cell subpopulation with a higher resistance to chemotherapy, which may have been key the recurrence of MEC.

## Introduction

Mucoepidermoid carcinoma (MEC) is common in human salivary glands. Poorly differentiated MEC is a lethal malignancy that readily invades nearby tissues and is likely to recur (1). Conventional surgery is the most common treatment method for MEC, however, often results in devastating functional and cosmetic consequences. In order to kill residual tumor cells and prevent the recurrence of MEC, chemotherapy is required following surgery. The chemotherapeutic agent, 5-fluorouracil (5-Fu), is commonly used; however, chemotherapy is unable to kill all of the remaining tumor cells or prevent the recurrence of MEC. The underlying mechanisms of MEC recurrence following chemotherapy have not yet been investigated.

Cancer stem-like (CSL)-cells are a rare population of cancer cells exhibiting stem cell properties, constituting a reservoir of self-sustaining cells with an exclusive ability to self-renew and maintain the tumor. CSL-cells were identified first in acute myeloid leukemia (2) followed by solid tumors and subsequently breast cancer in 2003 (3). CSL-cells have been isolated from a variety of human malignancies, including leukemia (2,4), breast cancer (3,5), brain tumors (6–8), hepatocellular carcinoma (9), pancreatic (10) and colorectal cancers (11,12), melanomas (13), prostate cancer (14) and bone sarcomas (15). CSL-cells are significant in tumor formation and growth (16–18). Potentially quiescent CSL-cells, which are vital and capable of repopulating under cancer therapies, may be a source of recurrence and drug resistance (3,19).

The present study aimed to investigate the effects of chemotherapy on the MC3 MEC cell line and the potential roles of CSL-cells in recurrent MEC following chemotherapy.

## Materials and methods

### Cell line and culture

The MC3 MEC cell line was provided and conserved at the State Key Laboratory of Oral Diseases, Sichuan University (Chengdu, China). The MC3 cells were maintained in a serum-containing medium composed of RPMI-1640 (Hyclone, Logan, UT, USA) and 10% fetal bovine serum (FBS; Gibco-BRL, Grand Island, NY, USA). The cells were incubated at 37°C in a 5% CO_2_ humidified atmosphere and passaged once every three days.

### MC3 cell culture in 5-Fu-containing medium

The MC3 cells were incubated in a serum-containing medium composed of RPMI-1640, 10% FBS and 1 peak plasma concentration of 100 μg/ml 5-Fu (20) at 37°C in a 5% CO_2_ humidified atmosphere for 24 h.

### Soft agarose assays of clone formation

The 5-Fu-treated and parent MC3 cells were seeded in 24-well plates. Low melting-point agarose (0.3 ml, 0.6%; Type VII, Sigma-Aldrich, St. Louis, MO, USA) was poured into each well and 0.3 ml (0.35%) agarose containing 100 cells was subsequently added to each well. The cells were incubated following the solidification of agarose at room temperature. The number of clones containing >50 cells was counted under a microscope after ten days and the cloning efficiency was calculated using the following formula: Colony formation rate (%) = no. of clones/no. of cells incubated × 100.

### MTT assay

The 5-Fu-treated and parent MC3 cells were seeded in 96-well plates, each well contained 2,000 cells and was cultured in complete RPMI-1640 medium with 10% FBS. The cell viability was measured using the MTT assay (Sigma-Aldrich). The optical density (OD) values were obtained using a microplate reader (ThermoElectron 3001 Varioskan Flash; USA) on days one, three, five, seven and nine.

### Quantitative polymerase chain reaction (qPCR)

qPCR was performed using the SYBR^®^ Green reporter to detect the expression of genes, cluster of differentiation (CD)44 and octamer-binding transcription factor 4 (Oct4). The primer sequences are summarized in Table I. The cells were harvested and RNA was extracted from the 5-Fu-treated and parent MC3 cells using TRIzol reagent (Invitrogen Life Technologies, Carlsbad, CA, USA), then reverse-transcribed into cDNA using PrimeScript RT reagent kit (Takara, Dalian, China) according to the manufacturer’s instructions. qPCR was performed according to the standard protocol of the SYBR Premix Ex Taq™ II kit (Takara) on an ABI 7300 Real Time PCR system (Applied Biosystems, Foster City, CA, USA). To quantify the changes in gene expression, the ΔΔCt method was used to calculate the relative fold changes following normalization using the internal reference gene, GAPDH.

### Immunocytochemistry

The 5-Fu-treated and parent MC3 cells were plated on glass coverslips at 37°C overnight, washed twice with PBS, and immunostained for CD44, Oct4 and the isotype control. The primary antibodies included rat monoclonal anti-CD44 (dilution 1:100; eBiosciences, San Diego, CA, USA) and rabbit monoclonal anti-Oct4 (dilution 1:50; Bioworld Technology, Minneapolis, MN, USA). The secondary antibodies included goat anti-rat IgG and goat anti-rabbit IgG (dilutions 1:50; Bios, Beijing, China). The intensity of 3,3′-diaminobenzidine was analyzed using the immunohistochemical Avidin Biotin Complex (ABC) method (15). Images were captured using a Nikon eclipse 80i microscope (Nikon Corp., Tokyo, Japan).

### Fluorescence-activated cell sorting (FACS) of CD44 and Oct4

The 5-Fu-treated and parent MC3 cells were trypsinized into solitary cell suspensions. The cells were counted, washed twice with PBS, resuspended in ice-cold PBS (supplemented with 2% FBS) and labeled with antibodies specific for human cells, such as rat monoclonal anti-CD44 antibody. The cells were incubated with their antibodies for 30 min at 4°C in the dark. The unbound antibodies were removed by washing twice with PBS. The fluorescein isothiocyanate (FITC)-labeled secondary antibody was added to the cell suspension and incubated for 30 min at 4°C in the dark. The cells were washed twice with PBS and FACS analysis (BD Biosciences, San Jose, CA, USA) was performed.

The 5-Fu-treated and parent MC3 cells were fixed and perforated, resuspended in ice-cold PBS and labeled with antibodies specific for human cells, such as rabbit monoclonal anti-Oct4 antibody. The cells were incubated with their antibodies for 30 min at 4°C in the dark. The unbound antibodies were removed by washing twice with PBS. The FITC-labeled secondary antibody was added to the cell suspension and incubated for 30 min at 4°C in the dark. The cells were washed twice with PBS and FACS analysis was performed.

### Culture of the cells in serum-free medium

The 5-Fu-treated and parent MC3 cells were washed three times with PBS to remove all traces of FBS. The cells were placed in serum-free Dulbecco’s modified Eagle’s medium (DMEM)/F12 (Hyclone), which was composed of 20 ng/ml basic fibroblast growth factor (PeproTech, Rocky Hill, NJ, USA), 20 ng/ml epidermal growth factor (PeproTech), 1 mg/ml insulin (Sigma-Aldrich) and 2% B27 (Invitrogen Life Technologies) at a density of 1×10^2^/ml. The cell suspensions (200 μl) were plated onto ultra-low attachment 96-well plates. The number of clones containing >50 cells was counted under a microscope on day seven and the cloning efficiency was calculated using the following formula: Colony formation rate (%) = no. of clones/no. of cells incubated ×100.

### FACS analysis of side population (SP) cells

SP cell analysis was based on a previously described method (21) with certain modifications. Briefly, cells were trypsinized and resuspended in PBS with 2% FBS at a density of 1×6^10^/ml. Verapamil (Sigma-Aldrich) at a final concentration of 50 μg/ml was added to the control group. After 10 min, 10 μg/ml Hoechst 33342 (Sigma-Aldrich) was added to the cell suspension, this was incubated in the dark for 90 min, centrifuged and resuspended in ice-cold PBS containing 2% FBS. Propidium iodine (2 μg/ml; Sigma-Aldrich) was added to separate the dead cells. Analysis and sorting were performed on a BD FACSAria.

### Statistical analysis

Statistical analyses were performed with SPSS software, version 11.5 (SPSS, Inc., Chicago, IL, USA). All quantified data present the means of at least three samples and error bars represent the standard deviation. Student’s t-test was used to determine the statistical differences between the experimental and control groups. P<0.05 was considered to indicate a statistically significant difference.

## Results

### 5-Fu-treated cells

The MC3 cells were exposed to 5-Fu for 24 h resulting in a large number of cell deaths. The dead cells were suspended in the medium and the surviving cells adhered to the plate wall. The viable cells were collected for subsequent experiments.

### 5-Fu-treated cells exhibit a higher cloning efficiency

**Figure 1:**
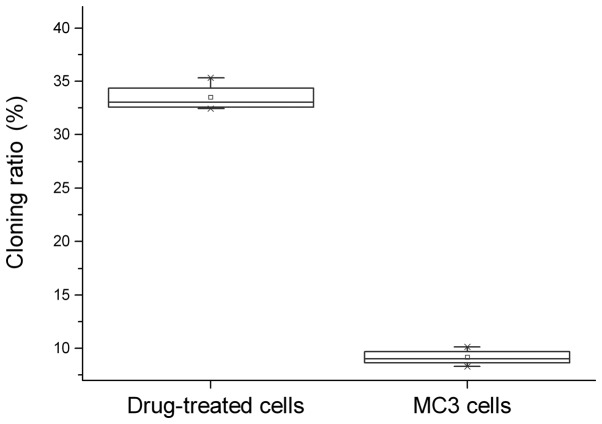
Cloning ratio of 5-Fu (drug)-treated and parent MC3 cells in soft agarose. The cloning ratio of 5-Fu-treated cells was significantly higher than the parent MC3 cells (P<0.05). 5-Fu, 5-fluorouracil.

The 5-Fu-treated and parent MC3 cells underwent the agarose colony formation experiments and showed that the cloning ratio of 5-Fu-treated cells (33.47±1.30%) was significantly higher compared with the parent MC3 cells (9.14±0.747%, P<0.05; Fig. 1).

### Growth curves of the cells

**Figure 2:**
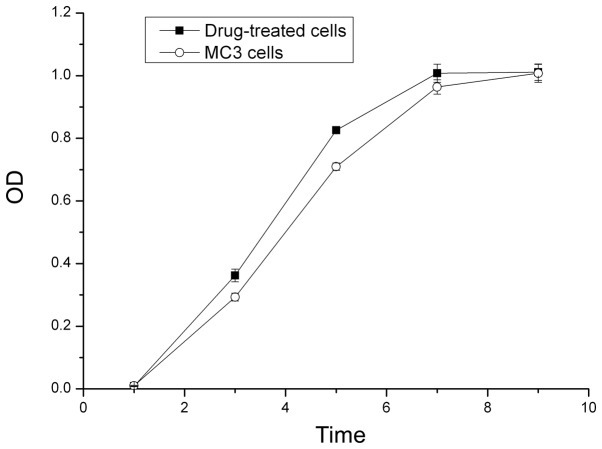
Growth curves of 5-Fu (drug)-treated cells and parent MC3 cells, the proliferative ability of 5-Fu-treated cells was significantly higher than the parent MC3 cells (P<0.05). 5-Fu, 5-fluorouracil; OD, optical density.

The OD values from the MTT assay were used to construct growth curves. The proliferative ability of the 5-Fu-treated cells was higher compared with the parent MC3 cells in the first seven days. The 5-Fu-treated cells reached the plateau phase on day seven, whereas the parent MC3 cells reached the plateau phase on day nine (P<0.05; Fig. 2).

### qPCR analysis

**Figure 3:**
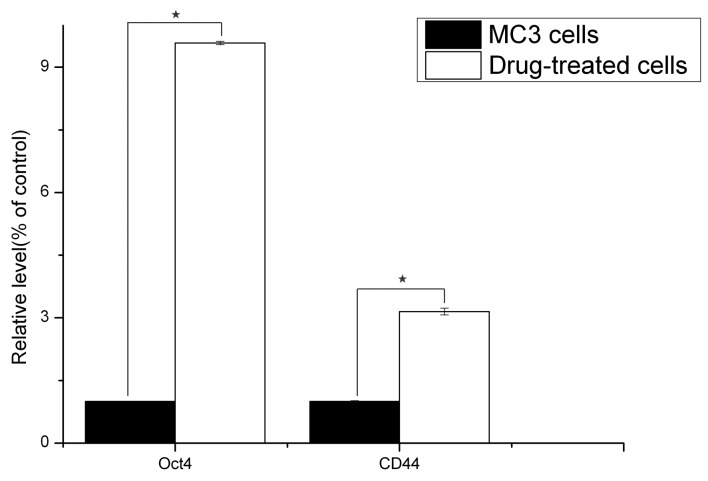
Gene expression examined by quantitative polymerase chain reaction. The expression of CD44 and Oct4 was statistically different between the 5-Fu (drug)-treated and parent MC3 cells (P<0.05). 5-Fu, 5-fluorouracil; Oct4, octamer-binding transcription factor 4; CD44, cluster of differentiation 44. ^*^P<0.05 parent MC3 cells vs. 5-Fu treated cells

The gene expression status of CD44 and Oct4 were compared between the 5-Fu-treated and the parent MC3 cells via qPCR. The results revealed that the reference gene, GAPDH, was stably expressed in all the samples, and CD44 and Oct4 were significantly expressed in the 5-Fu-treated cells compared with the parent MC3 cells (P<0.05; Fig. 3).

### CD44 and Oct4 protein expression

**Figure 4:**
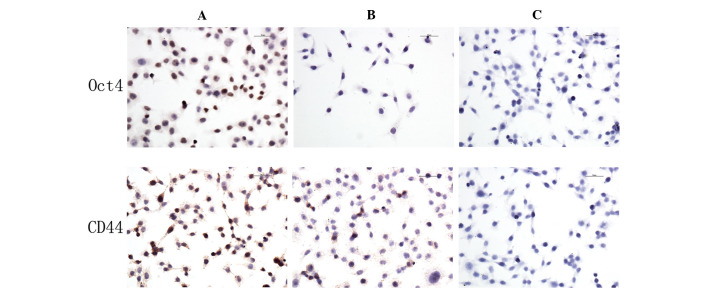
Immunocytochemistry assays of Oct4 and CD44 in (A) 5-Fu-treated and (B) parent MC3 cells. The expression of CD44 and Oct4 in the 5-Fu-treated cells was higher than in the parent MC3 cells. (C) Expression of Oct4 and CD44 in the isotype control. 5-Fu, 5-fluorouracil; Oct4, octamer-binding transcription factor 4; CD44, cluster of differentitation 44.

Immunocytochemistry assays were used to analyze the expression of CD44 and Oct4 in 5-Fu-treated and parent MC3 cells. The expression levels of CD44 and Oct4 in 5-Fu-treated cells were higher compared with the parent MC3 cells. CD44 was expressed in the cell membrane and cytoplasm, whereas Oct4 was expressed in the nucleus (Fig. 4).

**Figure 5:**
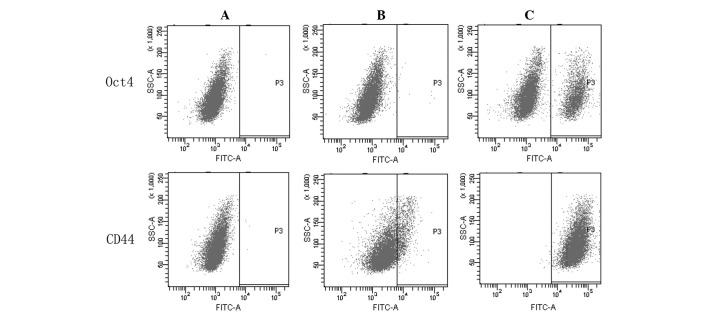
FACS analysis of Oct4 and CD44 in the (A) blank control, (B) parent MC3 and (C) 5-Fu-treated cells. The percentage of Oct4+ and CD44+ phenotype in the parent MC3 cells was 1.37±0.06 and 14.47±0.15%, respectively. The percentage of Oct4+ and CD44+ phenotype in the 5-Fu-treated cells was 14.60±0.36% and 99.50±0.30%, respectively. FACS, fluorescence-activated cell sorting; 5-Fu, 5-fluorouracil; Oct4, octamer-binding transcription factor 4; CD44, cluster of differentitation 44; FITC-A, fluorescein isothiocyanate labeled antibody.

Furthermore, the expression of CD44 and Oct4 was analyzed by FACS. According to three independent experiments, the expression levels of CD44 and Oct4 were 99.50±0.30 and 14.60±0.36%, respectively in the 5-Fu-treated cells, and 14.47±0.15 and 1.37±0.06%, respectively, in the MC3 cells (Fig. 5). The expression levels of CD44 and Oct4 were significantly different between the two cell populations (P<0.05).

### Spheroid cells in the serum-free medium

**Figure 6:**
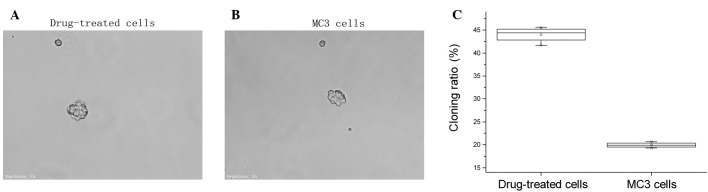
Spheroids of (A) 5-Fu (drug)-treated and (B) parent MC3 cells in serum-free medium. (C) The cloning (spheroids) ratio of 5-Fu-treated and MC3 cells was 44.02±1.71 and 19.94±0.57%, respectively (P<0.05). 5-Fu, 5-fluorouracil.

The 5-Fu-treated and parent MC3 cells that were incubated in serum-free medium for one day revealed multicellular spheroids. Spheroids were apparent following cell culture in serum-free medium for four days (Fig. 6). The number of cells in the spheroids gradually increased in a time-dependent manner and on day seven spherical bodies comprising of dozens of cells were observed. The number of spherical bodies increased by >20% following treatment with 5-Fu. When the spheroid cells were cultured in RPMI-1640 with 10% FBS they became adherent. These findings identified that under stem cell culture conditions, MC3 and 5-Fu-treated cells formed spheroids, and chemotherapy may improve the ratio of the formation of spheroids.

### SP cell assays

**Figure 7:**
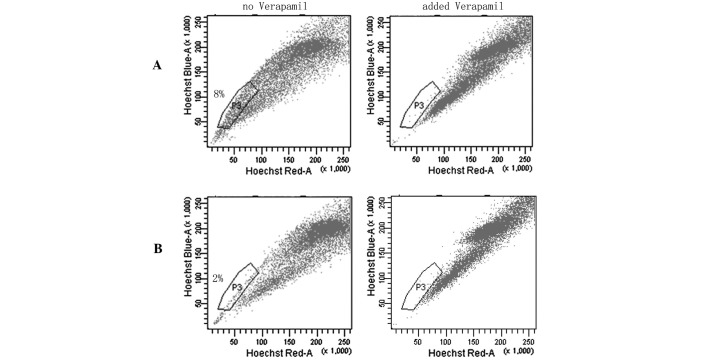
FACS analysis of (A) 5-Fu-treated and (B) parent MC3 cells stained with Hoechst 33342. The ratio of side population cells in the 5-Fu-treated and MC3 cells was 8.3±0.2 and 2.03±0.25%, respectively. FACS, fluorescence-activated cell sorting; 5-Fu, 5-fluorouracil.

SP flow cytometry has previously been used to enrich cancer stem cells (CSCs) from various cancer cell lines and primary tumors (22–24). SP cells do not fluoresce under the dual wavelength parameters of FACS as they are able to efflux Hoechst 33342 by adenosine triphospate-binding cassette transporters (21,25–29). In the SP assays, SP cells were located in the area of weak fluorescence and the ratio of SP to 5-Fu-treated cells was higher compared with MC3 cells. These data strongly indicated that chemotherapy may significantly increase the number of CSL-cells in MC3 cells (Fig. 7).

## Discussion

Previous studies have identified CSL-cells within tumors and that the injection of CSL-cells into nude mice induces the development of tumors. CSL-cells are considered to be comparable to normal tissue stem cells as they possess the ability to divide asymmetrically and symmetrically, and undergo multilineage differentiation (30,31). Similar to the activity of normal stem cells in the maintenance of tissue architecture, CSL-cells are regarded as a resource of tumor formation, progression, recurrence and drug resistance (11,32). CSL-cells are able to self-renew and differentiate into a diverse range of cells that form tumor masses (33,34). CSL-cells have a stronger resistance to traditional treatments, such as chemotherapy and radiation, compared with other types of tumor cells due to their high expression of drug resistant transporter proteins (such as ABC) (35–37), DNA repair enzymes (38,39) and anti-apoptotic proteins (40–42).

The present study indicated that the CSC phenotype may be induced by 5-Fu as cancer cells are able to acquire a stemness state, which is characterized by the increased stemness gene expression of Oct4. Oct4 is a typical stem-cell associated gene (43) and may be able to reprogram adult cells into induced pluripotent stem cells (iPS) (44,45). Despite the transcription factors of c-Myc, kruppel-like factor 4 and NANOG, Oct4 is an important gene as its expression is significant in the production of iPS (44,46,47). Previous studies identified a high expression of Oct4 in human embryonic stem cells compared with differentiated tissues and a high expression in CSL-cells compared with other types of cancer cells (18,48,49). In certain cell lines, the increased expression of Oct4 results in enhanced stemness and acquisition of a stem cell-like phenotype (50,51), which is associated with an increase in sphere formation and resistance to chemotherapy and radiotherapy. Knockdown of Oct4 may increase the sensitivity to chemotherapy and radiotherapy due to the restriction of the factors that lead to self-renewal. Therefore, the expression of Oct4 is important in the identification of CSL-cells.

As a type of transmembrane glycoprotein, CD44 is widely distributed on the cell surface of lymphocytes and fibroblasts (52). CD44 is predominantly involved in specific adhesion processes, such as cell-cell and cell-matrix. Thus, CD44 may be used as a surface marker of CSL-cells. In addition to breast cancer (3), CD44 was considered to be a CSL-cell marker in ovarian (53), prostate (54) and pancreatic cancer (10), and head and neck squamous cell carcinoma (55).

The present study demonstrated that the expression of Oct4 and CD44 increased following treatment with 5-Fu, particularly Oct4 expression in 5-Fu-treated cells, which was markedly higher compared with the parent MC3 cells. These findings were consistent with the increased stem cell-like phenotype, as the cloning ratio of the cells in the soft agarose increased from 9.14±0.747 to 33.47±1.30%. To examine this further, the 5-Fu-treated cells were cultured under stem cell culture conditions, which were selective for CSL-cell enrichment. The results indicated that chemotherapy was associated with a significant increase in sphere-formation ability, reflecting a greater self-renewal and proliferation ability of the 5-Fu-treated cells; furthermore, no difference in morphology was observed between the two types of spheroids. In addition, the 5-Fu-treated cells grew faster, reaching the plateau phase more rapidly than the parent MC3 cells in the MTT assays. These findings were consistent with previous studies, demonstrating that the drug resistance of tumor cells is associated with CSL-cells in tumors (17,56,57).

Over the past century, chemotherapy has been used extensively as a curative or adjuvant cancer treatment, particularly for metastatic tumors. However, the majority of human malignancies, including MEC, are resistant to this important therapeutic method. Resistance to chemotherapy is the primary obstacle for patient survival, particularly for those with metastatic tumors (58). In the present study, chemotherapy induced stem cell-like properties, such as sphere formation, clone formation and stemness-related gene expression, demonstrating that chemotherapy may enrich CSL-cells in the MC3 cell line. To further explore the number of CSL-cells in the 5-Fu-treated MC3 cells, flow cytometry using Hoechst 33342 dye exclusion was performed to isolate the SP cells that were enriched in CSCs. Notably, the drug-treated cells exhibited a higher percentage of SP cells compared with the parent MC3 cells; the CSC component in the MC3 cell line increased from 2 to 8% of the total cell population, indicating that they were more enriched for the CSC phenotype.

In conclusion, CSL-cells are considered to be a cause of tumors due to their similar characteristics to stem cells (self-renewal and multilineage differentiation). The present study indicated that 5-Fu may induce MC3 cells into a stem-like phenotype and that the remaining CSL-cells of MEC following chemotherapy were significant in tumor recurrence, as well as in promoting tumor survival. These findings demonstrated the mechanisms involved in the resistance of cancer cells to chemotherapy and implied that targeting CSL-cells may improve the efficacy of chemotherapy.

## Figures and Tables

**Table I:** Primer sequences for quantitative polymerase chain reaction.

Gene	Upstream primer	Downstream primer
CD44	5′-gagcagcacttcaggaggttaca-3′	5′-agtggtagcagggattctgtctg-3′
Oct4	5′-gcacaacgagaggattttgagg-3′	5′-agggaaagggaccgaggagta-3′
GAPDH	5′-ctttggtatcgtggaaggactc-3′	5′-gtagaggcagggatgatgttct-3′

CD44, cluster of differentiation 44; Oct4, octamer-binding transcription factor 4.
